# Frequency of the *Mycobacterium tuberculosis* RD^Rio^ genotype and its association with multidrug-resistant tuberculosis

**DOI:** 10.1186/s12879-019-4152-7

**Published:** 2019-06-25

**Authors:** Isabela Neves de Almeida, Sidra Ezidio Gonçalves Vasconcellos, Lida Jouca de Assis Figueredo, Nayanne Gama Teixeira Dantas, Cláudio José Augusto, João Paulo Amaral Hadaad, Philip Noel Suffys, Wânia da Silva Carvalho, Silvana Spíndola de Miranda

**Affiliations:** 1 Laboratório de Pesquisa em Micobactérias, Faculdade de Medicina, Universidade Federal de Minas Gerais (UFMG), Belo Horizonte, MG Brazil; 2 Laboratório de Biologia Molecular Aplicada a Micobactérias, Instituto Oswaldo Cruz, Fundação Oswaldo Cruz (FIOCRUZ), Rio de Janeiro, RJ Brazil; 3 Fundação Ezequiel Dias (FUNED), Belo Horizonte, MG Brazil; 4 Escola de Veterinária (UFMG), Belo Horizonte, MG Brazil; 5 Faculdade de Farmácia (UFMG), Belo Horizonte, MG Brazil

**Keywords:** Tuberculosis, *Mycobacterium tuberculosis*, Multidrug-resistant tuberculosis, Genotype, Single nucleotide polymorphisms

## Abstract

**Background:**

In recent decades, *Mycobacterium tuberculosis* with the RD^Rio^ genotype, frequently isolated from tuberculosis patients in Rio de Janeiro, has become part of the Latin American – Mediterranean (LAM) family and has been associated with multidrug-resistant tuberculosis (MDR-TB). The aim of this study was to investigate the frequency of *M. tuberculosis* RD^Rio^ in the state of Minas Gerais, Brazil, and its relationship with MDR-TB.

**Methods:**

For convenience, 172 susceptible and 63 MDR *M. tuberculosis* isolates were taken from pulmonary samples from patients diagnosed between January 2007 and December 2011. The DNA extracted from these isolates was analyzed by spoligotyping, PCR-RFLP to characterize *fbpC*^*103*^*/*Ag85C103, multiplex PCR to detect RD^Rio^ and RD174, and MIRU-VNTR *24 loci*.

**Results:**

Among the 235 isolates, the RD^Rio^ pattern was identified in 122 (51.9%) isolates (IC 0.45–0.58), with 100 (42.5%) wild-type and 13 (5.5%) mixed pattern isolates, whereas RD174 was identified in 93 of the 122 RD^Rio^ positive samples (76.3%). The LAM family and the LAM9 lineage were the most frequently identified among the isolates in this study. Among the 63 MDR isolates*,* 41 (65.1%) were RD^Rio^ and 28 (44.4%) RD174.

**Conclusion:**

The association of both deletions with MDR proved to be statistically significant, corroborating the few reports that have associated RD^Rio^ with MDR.

**Electronic supplementary material:**

The online version of this article (10.1186/s12879-019-4152-7) contains supplementary material, which is available to authorized users.

## Background

In 2017, 10 million new cases of tuberculosis (TB) were reported worldwide, with 558,000 new cases of rifampicin-resistant tuberculosis (RR-TB). Among RR-TB cases, an estimated 82% had multidrug-resistant TB (MDR-TB), and in Brazil in the same year, there were 2000 cases of MDR/RR-TB among pulmonary TB cases [1]. *Mycobacterium tuberculosis* (*M. tuberculosis*) is a human pathogen that undergoes clonal evolution, resulting in divergent lineages associated with specific geographic regions, and possibly with different human ethnic populations [2]. These varied lineages present with biological differences regarding transmissibility [3].

Molecular analyses based on specific genetic markers enable the rapid identification of different species and sublineages, an important tool for studying the evolution and transmission of *M. tuberculosis* [4]. The marker used to characterize the Latin American - Mediterranean (LAM) family is the single nucleotide polymorphism (SNP) *fbpC*^*103*^*/*Ag85C103, which is considered important for identification due to its high specificity. In addition, regions of difference such as RD^Rio^ and RD174 are also lineage specific, and the latter has been associated with higher levels of transmissibility [4].

The LAM family accounts for approximately 15% of the global burden of TB, and is present in 46% of the isolates that have been analyzed through genotyping in Brazil [4]. The LAM9 lineage in particular represents 10.2% of the isolates of *M. tuberculosis* on the American continent [5]. In 2007, Lazzarini et al. [6] described a genotype of *M. tuberculosis* called RD^Rio^, which is exclusively found as a sublineage derived from the LAM family [6]. It is believed that this genotype originated from a progenitor LAM9 and gave rise to the sublineages LAM1, LAM2, LAM4, and LAM5, as documented by the successive loss of spacers in spoligotype patterns [6–8].

The *M. tuberculosis* RD^Rio^ genotype contains a 26.3 kb deletion that causes the loss and modification of 10 genes, including two PPE (*Proline-glutamic Acid Proteins*) genes which encode specific proteins important for immunomodulation [5]. This sublineage has been isolated in several places in Brazil and other countries, and has been associated with higher levels of transmission, such as that found in MDR-TB [2, 3, 7].

Another important molecular technique that allows for the phylogenetic study of the *M. tuberculosis* complex is the Variable Number Tandem Repeat – Mycobaterial Interspersed Repetitive Unit (MIRU-VNTR 24 loci) based genotyping tool. This, together with spoligotyping, resulted in the construction of large genotypic databases that allowed for the phylogenetic analysis and study on the global distribution of *M. tuberculosis* [9]. Genotyping by means of MIRU-VNTR 24 loci also enables the study of the epidemiologically significant clonal diversity of *M. tuberculosis* strains, which is useful for exploring internal phylogenetic ramifications [7, 9–11]. There are some studies that describe the frequency of *M. tuberculosis* RD^Rio^ in certain populations, but data evaluating the significance of this relationship by means of specific statistical tests on the co-occurrence of this genotype with MDR-TB are scarce, and no such data is available for the state of Minas Gerais [2, 3, 12]. In this context, the aim of this study was to evaluate the frequency of *M. tuberculosis* RD^Rio^ isolation in this region of Brazil, and its relationship with MDR-TB and other genetic markers affecting the drug susceptibility profile.

## Methods

### Study design

For convenience, 172 susceptible and 63 MDR-TB (strains defined as drug resistant to at least isoniazid and rifampin) *M. tuberculosis* isolates were collected. Isolates with single-drug resistance were not included.

The isolates were obtained from pulmonary samples (sputum and bronchoalveolar lavage) from patients diagnosed between January 2007 and December 2011, in Minas Gerais.

In this state, the mean MDR-TB rate was 0.2% among clinical TB cases between 2002 and 2009 [13], while after this period MDR-TB detection rates increased due to the expansion of culture and the susceptibility testing. In the present study, the identification of *M. tuberculosis* was performed by phenotypic testing [14], while drug susceptibility testing was conducted using the BACTEC™ MGIT™ 960 system (Becton Dickinson®), according to manufacturer’s instructions [15], in the Main Public Health Laboratory of the state of Minas Gerais located in the Octavio Magalhães Institute of the Ezequiel Dias Foundation in Belo Horizonte. All 235 clinical isolates were analyzed by means of the molecular tests described below, the results of which are included in the statistical analyses.

### DNA extraction

The genomic DNA of *M. tuberculosis* was extracted from subcultured colonies in Lowenstein-Jensen solid medium using 10% Cetyltrimethylammonium Bromide (CTAB) as described by Dantas et al. [16] in 2015. The extracted DNA was used for the techniques described below. The experiments were performed in duplicate, with the exception of spoligotyping and MIRU-VNTR 24 loci.

### Multiplex PCR - RD^Rio^

The detection of the RD^Rio^ pattern was performed by multiplex PCR, using the following set of primers: BridgeRD^Rio^F, BridgeRD^Rio^R, IS1561F, and IS161R, and was confirmed by the presence or absence of 1175 and/or 530 bp fragments. The RD^Rio^ pattern presents a 1175 bp fragment, while the wild-type (WT) produces a 530 bp fragment [4, 6, 7].

### Multiplex PCR - RD174

To perform amplification, we used the PCR protocol describe by Gibson et al. [4] in 2008, and adapted by Vasconcelos et al. [7] in 2014. Briefly, we used a primer for each of the following: RD174 F, RD174Fi, and RD174 R. The isolates showing an intact RD174 region (WT) produced 300 bp fragments, while the RD174 deletion presented as 500 bp fragments [4, 7].

### Spoligotyping

Spoligotyping was performed using the Beamedex® microsphere technique in a Luminex-Bioplex-BioRad 200® system, as developed in the “Institut de Genétique et Microbiologi e Université Paris-Sud”, and following the protocol described by Zhang et al. (2010) [17].

### PCR-RFLP fbpC^103^/Ag85C103

The SNP *fbp*C^103^,or Ag85C103, was described as a specific marker for the LAM lineage by Gibson et al. [4] in 2008. The PCR protocol used in this study was adapted as described by Vasconcelos et al. [7] in 2014. To perform the amplification, we used this set of primers: fbpC103 F and *fbpC*^*103*^ R. The amplified products (519 bp) were analyzed on 2% agarose gel in 1× TBE. After this step, the enzymatic digestion was performed by restriction enzyme *Mn*II (New England BioLabs Inc. USA). The *Mn*II enzyme produces three restriction fragments in the amplified product: 365 bp, 96 bp, and 48 bp. The presence of SNP (G309A) (LAM) results in the loss of one of the three restriction sites [4, 7].

### MIRU-VNTR 24 loci

The MIRU-VNTR 24 loci was performed according to the protocol described by Supply et al. [9] in 2006. This procedure used a monoplex PCR, using the fragments revealed by electrophoresis, in 2% agarose gel. In the construction of the dendogram, the Neighbor-Joining (NJ) algorithm was used to analyze the categorical data.

### Statistical analyses

Associations were calculated using the chi-square and Fisher’s tests, and performed by STATA 12 software (Copyright 1985–2015 StataCorpLP©, USA).

### Phylogenetic analyses

The analyses were performed using these free sites: the SITVIT website *http://www.pasteur-guadeloupe.fr:8081/SITVIT_ONLINE/* and the MIRU-VNTRplus Server Policy *http://www.miru-vntrplus.org/MIRU/index.faces*, as well as with Bionumerics 7.0©Applied Math software (Sint maertens, Latem, Belgium).

## Results

### *Mycobacterium tuberculosis* RD^Rio^ - frequency and association with MDR-TB

**Fig. 1:**
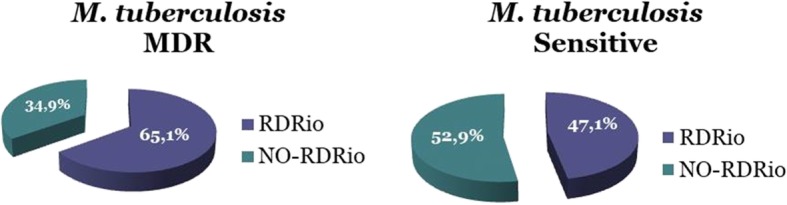
*Mycobacterium tuberculosis* RD^Rio^ and its association with MDR-TB. This grafics shows the percentages of RD^Rio^ and NO-RD^Rio^ in susceptible and MDR-TB clinical isolates. It’s important to highlight the relationship between the RD^Rio^ sublineage and MDR-TB was significant (*p* < 0.001)

Among the 235 *M. tuberculosis* isolates, 122 (51.9%) were identified as RD^Rio^ (IC 0.45–0.58), 100 (42.5%) as NO-RD^Rio^, and 13 (5.5%) as mixed pattern. The relationship between the RD^Rio^ sublineage and MDR-TB was significant (*p* < 0.001, chi-square test). The percentages of RD^Rio^ and NO-RD^Rio^ in susceptible and MDR-TB clinical isolates are shown in Fig. 1.

Of the 122 *M. tuberculosis* RD^Rio^, 116 (95.1%) were identified as LAM, while the remaining six (4.9%) were identified as WT based on the Ag85C103 SNP, and this relationship was significant (*p* < 0.000, chi-square test, and *p* < 0.000, Fisher’s exact test). The genetic profiles of these six RD^Rio^, classified as WT by means of AG85C103 SNP, and found after analyses performed by spoligotyping and MIRU-VNTR 24 loci, are described in Table 1.

**Table 1:** Genotypic profile of the *Mycobacterium tuberculosis* RD^Rio^ isolates, classified as wild type through the SNP AG85

*M. tuberculosis* RD^Rio^		
Spoligotyping	MIRU-VNTR 24 loci	SNP AG85
T1	Delhi/CAS	WT
T1	Haarlem	WT
H3	Haarlem	WT
LAM 1	Cameroon	WT
LAM 2	LAM	WT
Unknown	LAM	WT

Legend: *WT: Wild Type

**Fig. 2:**
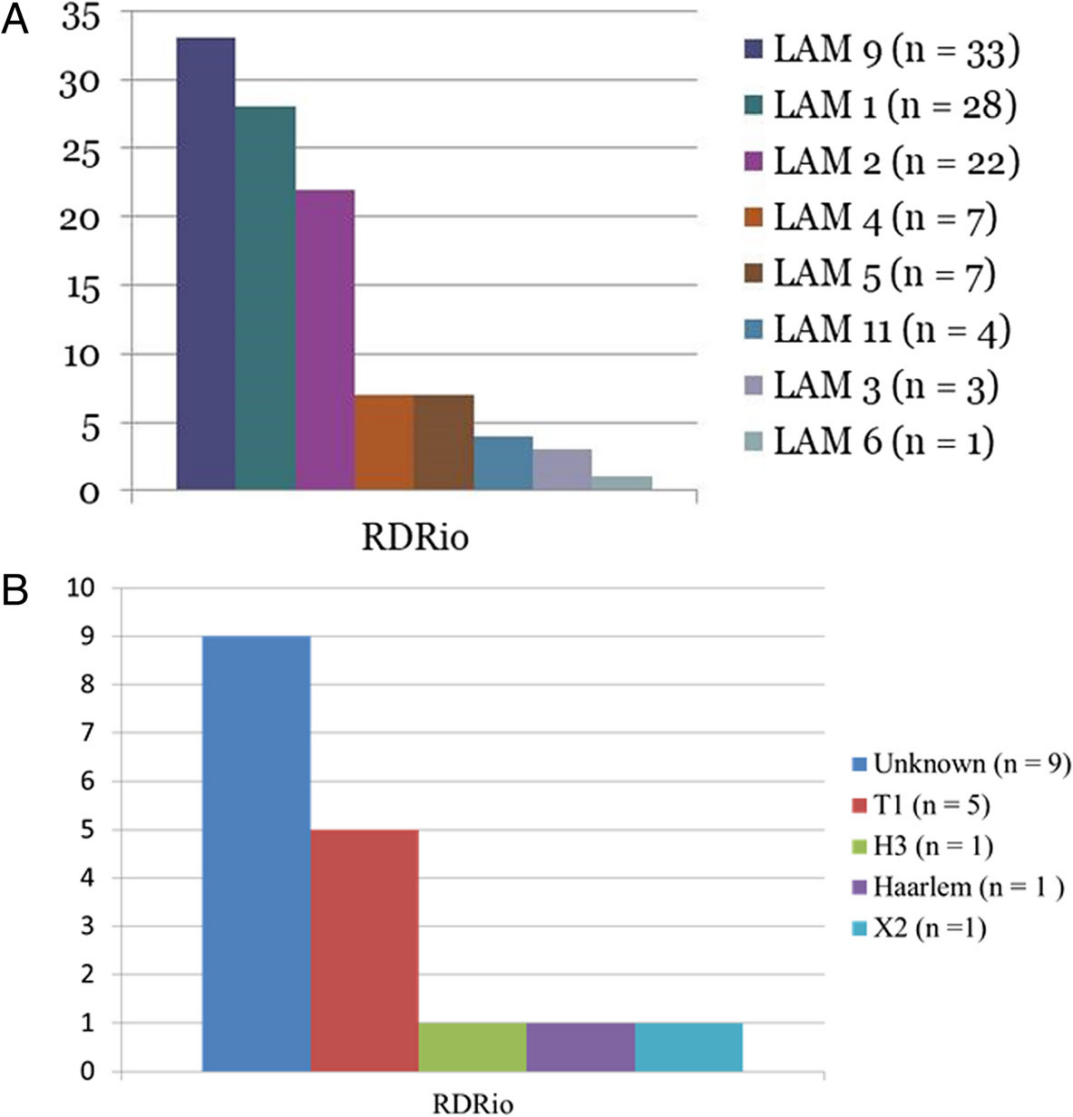
The spoligotyping of *M. tuberculosis* RD^Rio^. A = The distribuition of RD^Rio^ spoligotyping’s pattern of the LAM family. B = The distribuition of RD^Rio^ spoligotyping’s pattern of the NO-LAM families

The spoligotyping show that the majority of the RD^Rio^ isolates proved to be LAM. The RD^Rio^ spoligotyping’s patterns are shown in Fig. 2. The distribution of profiles found among drug resistant and susceptible isolates is shown in Additional file 1. The spoligotyping classifications for all 172 sensitive and 63 MDR isolates are described in Table 2.

**Table 2:** Classification of *Mycobacterium tuberculosis* strains in Minas Gerais according to spoligotyping

	*M. tuberculosis*	
Lineages	Sensitive _*n* = 172_ (73%)	MDR _*n* = 63_ (27%)
LAM 9	34 (19.7)	18 (28.5)
LAM 1	20 (11.6)	13 (20.6)
LAM 2	18 (10.5)	4 (6.3)
LAM 3	12 (6.9)	2 (3.7)
LAM 4	6 (3.5)	2 (3.7)
LAM 5	5 (2.9)	3 (4.7)
LAM 6	2 (1.2)	–
LAM 11-ZWE	4 (2.3)	–
T1	16 (9.3)	9 (14.2)
T2	2 (1.2)	2 (3.7)
T3	2 (1.2)	–
T4-CEU	2 (1.2)	–
T5-Madrid2	2 (1.2)	–
T3/T2	1 (1.6)	–
H1	4 (2.3)	1 (1.5)
H2	2 (1.2)	–
H3	10 (5.8)	1 (1.5)
Haarlem	2 (1.2)	–
X2	9 (5.2)	2 (3.7)
Orphan	4 (2.3)	–
*Unkonwn*	18 (10.5)	7 (11.1)

### Identification of the RD174 pattern

Among the 235 isolates of *M. tuberculosis*, the RD174 pattern was identified in 98 (41.7%), while 111 (47.2%) were WT and 26 (11.1%) presented a mixed pattern (RD174/WT).

Of the 98 isolates that presented as RD174, 93/98 (94.9%) were identified as RD^Rio^ (*p* < 0.000, chi-square test).

Among the 63 resistant *M. tuberculosis*, 28 (44.4%) presented the RD174 pattern, while among the 172 sensitive isolates 90 (52.3%) presented the WT pattern. The relationship between the RD174 pattern and TB drug resistance was significant, as was relationship between the WT pattern and drug sensitivity (*p* < 0.001, chi-square test, and *p* < 0.002, Fisher’s exact test).

### Identification of the Ag85C103 SNP and comparison with spoligotyping

**Fig. 3:**
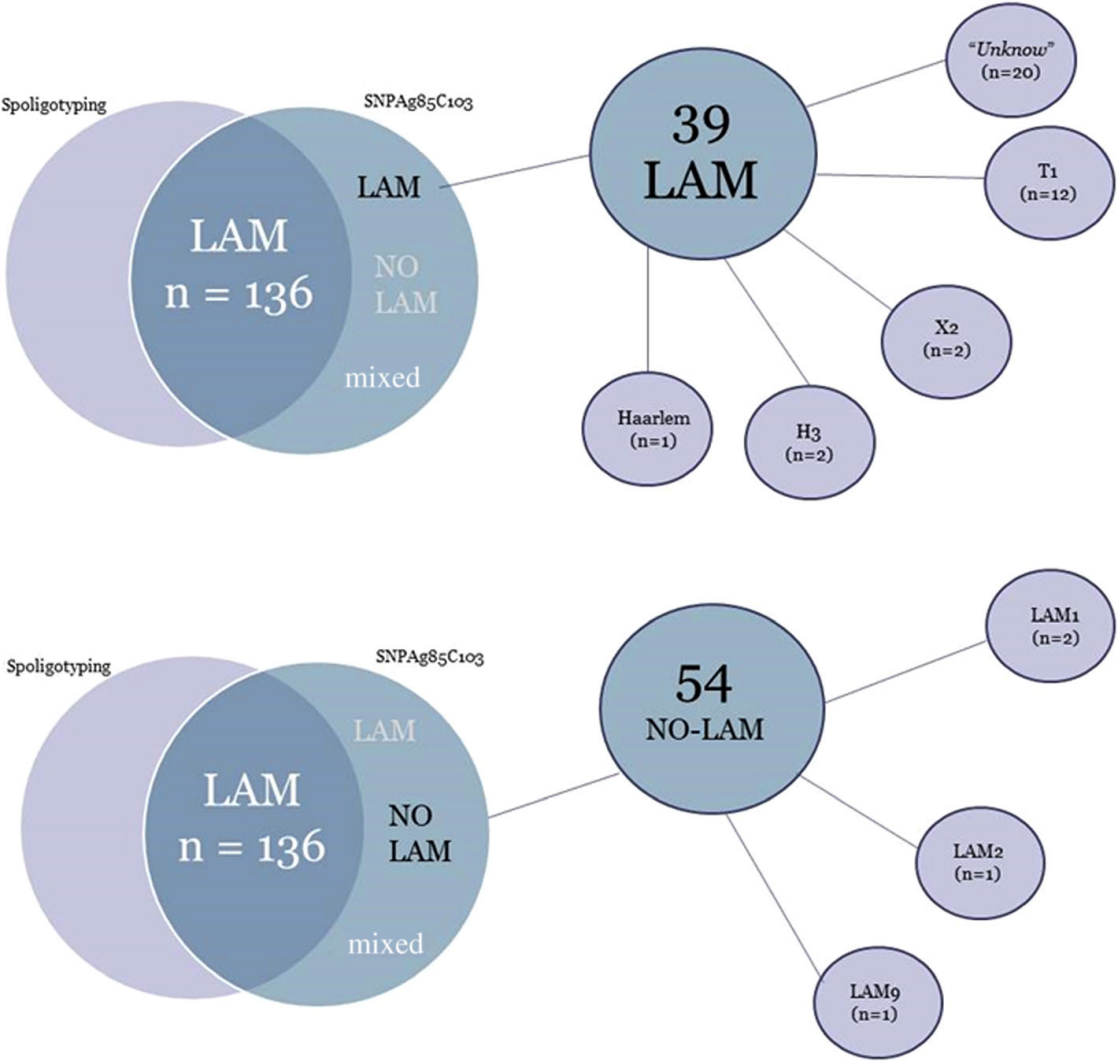
The relationship between Ag85C103 SNP and spoligotyping. Purple circle – spoligotyping, Blue circle **-** Ag85C103

Of the 235 *M. tuberculosis* identified by the Ag85C103 SNP, 175 (74.4%) were classified as LAM, 54 (23%) as non-LAM, and six (2.5%) presented a mixed pattern (SNP/nonSNP). The profile of these isolates is described in Table 3, of which only one presented a mixed pattern concurrent with the other markers (RD^Rio^ and RD174). The relationship between these and the spoligotyping results is shown in Fig. 3.

**Table 3:** Genotypic profile of *M. tuberculosis* exhibiting a mixed pattern in AG85C103 SNP analysis

*M. tuberculosis* AG85C103 SNP mixed pattern		
Spoligotyping	MIRU-VNTR 24 loci	RD^Rio^
T1	UgandaI	NO RD^Rio^
T3	X	NO RD^Rio^
LAM 9	Cameroon	Mixed
X2	X	NO RD^Rio^
T5-Madrid2	Cameroon	NO RD^Rio^
H3	Haarlem	NO RD^Rio^

Analysis of the Ag85C103 SNP detected a higher frequency of LAM genotypes than spoligotyping, and this difference proved to be significant (*p* < 0.000, chi-square test, and *p* < 0.003, Fisher’s exact test).

No significant difference was observed between susceptible and resistant *M. tuberculosis* in isolates characterized as LAM by Ag85C103 (*p* < 0.309, chi-square test, and *p* < 0.428, Fisher’s exact test).

### MIRU-VNTR 24 loci

**Fig. 4:**
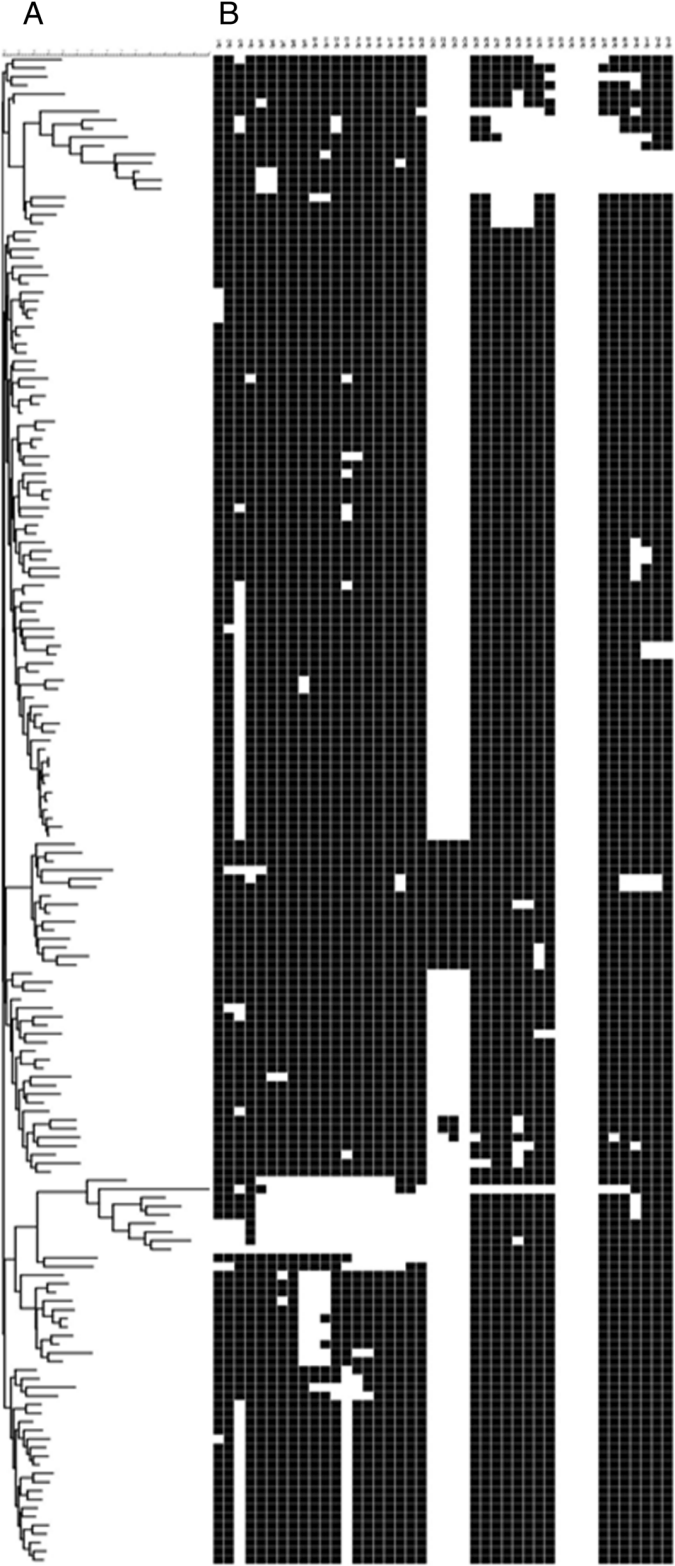
Dendogram of association by MIRU-VNTR 24 loci, spoligotyping, and RD174. A = Dendogram with association of all strains with the pattern RD174 and this respective MIRU-VNTR 24 loci. B = The spoligotyping of all strains with the pattern RD174

**Fig. 5:**
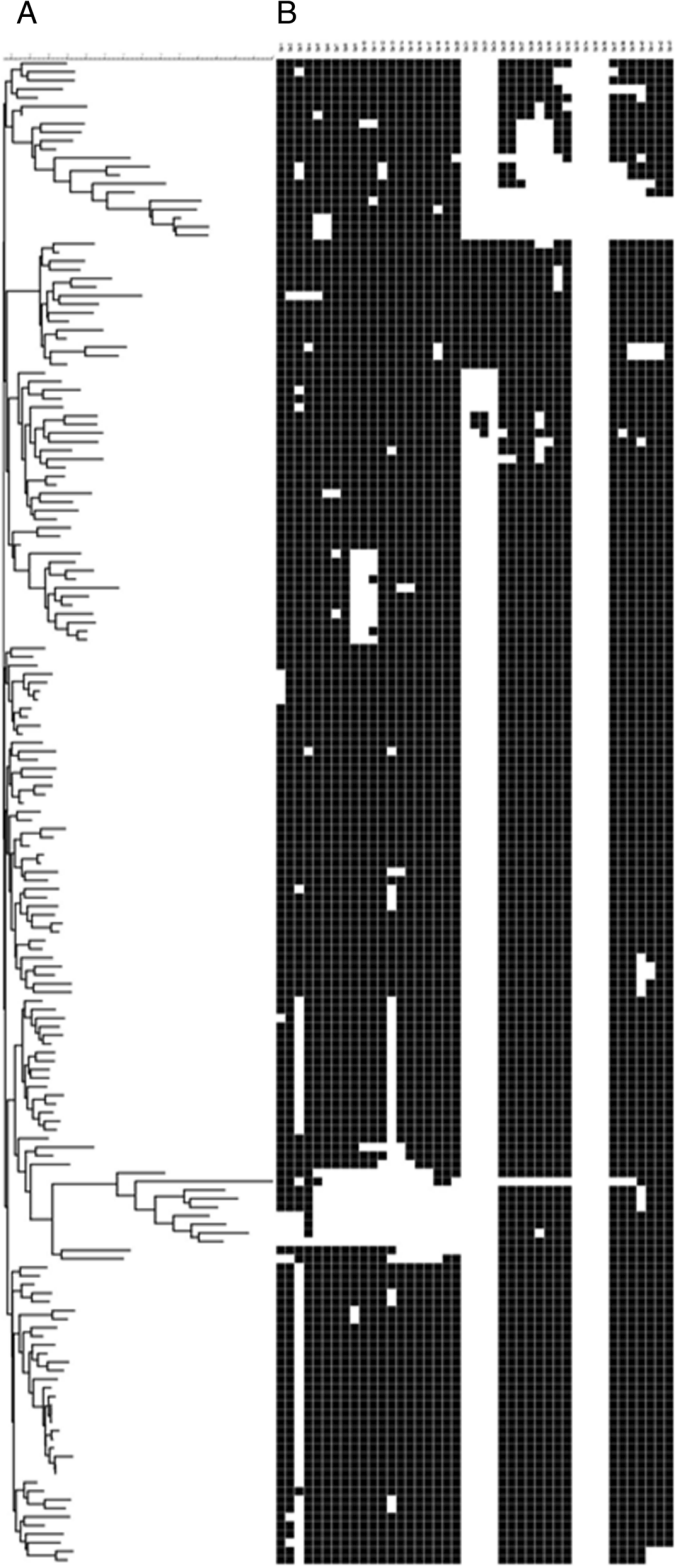
Dendogram of association by MIRU-VNTR 24 loci, spoligotyping, and RD^Rio^. A = Dendogram with association of all strains with the pattern RD^Rio^ and this respective MIRU-VNTR 24 loci. B = The spoligotyping of all strains with the pattern RD^Rio^

Among the 235 isolates, MIRU-VNTR 24 loci typing revealed the following families: LAM, 136 (69.4%); Haarlem, 36 (15.3%); Cameroon, 13 (5.5%); X, 10 (4.3%); Unganda I, 7 (3%); Delhi/CAS, 4 (1.7%); one from Ghana (0.4%), and one from a NEW-1 type. A UPGMA-based dendrogram demonstrates the four clusters of the RD^Rio^ sublineage (Figs. 4 and 5).

## Discussion

The present study demonstrates a significant relationship between the RD^Rio^ sublineage and MDR-TB, and is the first study of its kind in the state of Minas Gerais. In contrast to previous studies that only evaluated the frequency of this sublineage, it is important to note in the present study that we also evaluated the relationship of not only RD^Rio^, but also of other phylogenetic markers (RD174, Ag85C103 SNP), with their susceptibility or resistance to anti-TB drugs.

Lazzarini et al. [6] in 2007 suggested that the *M. tuberculosis* genotype RD^Rio^ originated from a common progenitor, since IS1561 deletion was also found in other countries. In addition, besides their capacity for progressive primary TB, isolates with this genotype have been associated with the development of the MDR phenotype [4, 6, 7].

The high frequency of RD^Rio^ observed in this study population may well be due to the predominance of the LAM family and the LAM9 lineage, since they are the most common progenitors of this sublineage [3, 4, 6, 7]. We also observed a statistically significant predominance of the RD^Rio^ sublineage among MDR isolates, as compared to sensitive isolates. This result is consistent with data obtained in Porto Alegre, where RD^Rio^ was observed in 56 of the 115 MDR isolates, accounting for almost half of the resistant isolate cases [12]. In a study performed in Portugal, the RD^Rio^ frequency was 60% among MDR isolates [3], while in the United States and Spain the RD^Rio^ frequency in MDR isolates was no higher than in sensitive isolates, but was identified among both MDR and isoniazid monoresistant isolates [2, 12]. In those same studies in Spain and the United States, RD^Rio^ was found in a higher proportion in Hispanic patients [2, 18]. Lazzarini et al. [19] analyzed isolates from several parts of Brazil in 2008, and suggested that the sublineage RD^Rio^ LAM could cause more severe disease (cavitary lung lesions), and most likely contributes to the transmission of TB among certain ethnic populations [6, 12, 19].

RD^Rio^ may have some biological advantage over other genotypes due to the deletion of two PPE genes (PPE55 and PPE56), and may minimize the host’s immunological recognition leading to increased virulence and/or transmissibility [4, 12].

In a study that evaluated RD^Rio^ in TB contacts in Gambia [20], RD174 was the second most frequent marker found, suggesting a certain association with the RD^Rio^ deletion. The most frequent marker was RD702, but this was related to the transmission of *M. africanum* [20].

In our study, although RD174 was identified in most of the isolates with RD^Rio^ (94.9%), and though the relationship between these markers was significant (*p* = 0.000), their correlation is not absolute. Therefore, when analyzing only RD174, one can overestimate the RD^Rio^ frequency in the present population, as described by another author that analyzed isolates from Rio de Janeiro [7]. This data contradicts a study by Gibson et al. [4] in 2008, who considered RD174 an absolute marker for RD^Rio^. One explanation for this difference may well be the local features of sample selection.

As shown in this work, spoligotyping exhibits limitations in differentiating Euro American families in populations that predominate with the LAM, H, and T families, and the fact that we found six RD^Rio^ isolates characterized as Non-LAM by spoligotyping may be due to these limitation [4, 7, 8]. The difficulty in differentiating these families comes from the large number of IS6110 of these strains produce, with many variations in the “Direct Repeat” (DR) locus, which gives rise to several profiles not identified by this technique (*Unkonwn pattern*), or with a high degree of homoplasy between the spacers that define different families [15, 16]. In this context, the Ag85C103 SNP is particularly important as a specific additional marker used to identify the frequency of the LAM family in a population [2, 4].

One limitation of this study is that since the RD^Rio^ sublineage could not be correlated with patients’ clinical data, it was impossible to associate RD^Rio^ strains with a prognosis for severe forms of TB. Another limitation is that we did not perform an entire genome analysis of the present strain’s population, and it has been shown that differences occur between this level of genotyping and 24 MIRU-VNTR typing and lineage definition. This finding would be particularly interesting for better characterizing the isolates that present with either RD^Rio^ or RD174, but not both. However, we must emphasize that the methods employed in this study are feasibly useful in places with few resources. In contrast, though WGS reduces the processing time, it is still an expensive technology that demands heavy bioinformatics to analyze the data. The results of this study were obtained through simple molecular biology techniques and may be important for the control and monitoring of TB and TBMDR.

## Conclusions

In conclusion, most of the isolates of *M. tuberculosis* in Minas Gerais belong to the LAM family, the LAM9 lineage, and the RD^Rio^ sublineage. Both *M. tuberculosis* RD^Rio^ and RD174 isolates positively correlated with MDR-TB. Because Brazil has a vast territory with considerable demographic and economic differences, further studies of RD^Rio^ in other regions are required*.*

## Additional file

Additional file 1: The Genetic profile of *Mycobacterium tuberculosis* RD^Rio^ in Minas Gerais as defined by spoligotyping (DOCX 23 kb)

## Data Availability

The analyses for objectives of this study were conducted using STATA 12 software (Copyright 1985–2015 StataCorpLP©, USA). The data that support the findings of this study are available from Silvana Spindola de Miranda upon reasonable request by qualified researchers.

## Abbreviations

LAM: Latin American – Mediterranean
*M. tuberculosis*: *Mycobacterium tuberculosis*
MDR-TB: Multidrug-resistant tuberculosis
MIRU-VNTR: Variable Number Tandem Repeat – Mycobaterial Interspersed Repetitive Unit
PPE: Proline-glutamic Acid Proteins
RR-TB: Rifampicin-resistant tuberculosis
SNP: Single Nucleotide Polymorphism
TB: Tuberculosis
WT: Wild-type
