# Supplementary material for: Frequency of the Mycobacterium tuberculosis RDRio genotype and its association with multidrug-resistant tuberculosis
Source: BMC Infect Dis. 2019 Jun 25;19:556. doi: 10.1186/s12879-019-4152-7 (PMC6593491; doi:10.1186/s12879-019-4152-7)
Supplement: Supplementary file 1 — The Genetic profile of Mycobacterium tuberculosis RDRio in Minas Gerais as defined by spoligotyping (DOCX 23 kb) [file 12879_2019_4152_MOESM1_ESM.docx]

**The Genetic profile of *Mycobacterium tuberculosis* RD^Rio^ in Minas Gerais as defined by spoligotyping**

| Lineage | | SIT | | Octal | | | RD174 | | SNPAG85C^103^ | MDR | Sensitive |
| --- | --- | --- | --- | --- | --- | --- | --- | --- | --- | --- | --- |
| LAM 9 | | 42 | | 777777607760771 | | | + | | LAM | 9 | 11 |
|  |  | | | |  |  | |  |  |  |  |
|  | |  | |  | | | +/- | | LAM | 2 | 5 |
|  | |  | |  | | | - | | LAM | 0 | 1 |
| LAM 9 | | 177 | | 377777607760771 | | | + | | LAM | 1 | 3 |
|  | | | | | | |  | |  |  |  |
| LAM 9 | | 2070 | | 777777637760771 | | | +/- | | LAM | 1 | 0 |
|  | | | | | | |  | |  |  |  |
| LAM 1 | | 20 | | 677777607760771 | | | + | | LAM | 7 | 11 |
|  | | | | | | |  | |  |  |  |
|  | |  | |  | | | +/- | | LAM | 4 | 0 |
| LAM 1 | | 753 | | 477777607760771 | | | +/- | | LAM | 1 | 0 |
|  | | | | | | | - | | LAM | 1 | 0 |
| LAM 1 | | 2536 | | 676777607760771 | | | + | | LAM | 0 | 2 |
|  | | | | | | |  | |  |  |  |
| LAM 1 | | 2522 | | 677777607760740 | | | + | | LAM | 0 | 2 |
|  | | | | | | |  | |  |  |  |
| LAM2 | | 17 | | 677737607760771 | | | + | | LAM | 2 | 16 |
|  | | | | | | |  | |  |  |  |
|  | |  | |  | | | + | | NO-LAM | 0 | 1 |
| LAM2 | | 1694 | | 277737607760771 | | | + | | LAM | 0 | 1 |
|  | | | | | | |  | |  |  |  |
| LAM3 | | 1491 | | 740000007760731 | | | + | | LAM | 1 | 0 |
|  | | | | | | |  | |  |  |  |
| LAM3 | | 33 | | 776177607760771 | | | - | | LAM | 0 | 1 |
|  | | | | | | |  | |  |  |  |
| LAM3 | | ORPHAN | | 772177607760771 | | | + | | LAM | 0 | 1 |
|  | | | | | | |  | |  |  |  |
| LAM5 | | 216 | | 777717607760771 | | | + | | LAM | 1 | 1 |
|  | | | | | | |  | |  |  |  |
| LAM5 | | 93 | | 777737607760771 | | | + | | LAM | 1 | 0 |
|  | | | | | | | +/- | | LAM | 0 | 1 |
| LAM5 | | 1693 | | 737737607760771 | | | + | | LAM | 0 | 1 |
|  | | | | | | |  | |  |  |  |
| LAM5 | | 440 | | 777607607760771 | | | +/- | | LAM | 0 | 1 |
|  | | | | | | |  | |  |  |  |
| LAM5 | | ORPHAN | | 777017607760771 | | | +/- | | LAM | 1 | 0 |
|  | | | | | | |  | |  |  |  |
| LAM4 | | 60 | | 777777607760731 | | | + | | LAM | 1 | 2 |
|  | | | | | | |  | |  |  |  |
| LAM4 | | 1530 | | 777777607760711 | | | + | | LAM | 1 | 1 |
|  | | | | | | |  | |  |  |  |
| LAM4 | | ORPHAN | | 777777607740031 | | | + | | LAM | 0 | 1 |
|  | | | | | | |  | |  |  |  |
| LAM6 | | ORPHAN | | 757777607560771 | | | + | | LAM | 0 | 1 |
|  | | | | | | |  | |  |  |  |
| LAM 11 -ZWE | | 59 | | 777777606060771 | | | + | | LAM | 0 | 3 |
|  | | | | | | |  | |  |  |  |
| LAM 11 -ZWE | | ORPHAN | | 777177606060771 | | | + | | LAM | 0 | 1 |
|  | | | | | | |  | |  |  |  |
| X2 | | 137 | | 777776777760601 | | | + | | LAM | 0 | 1 |
|  | | | | | | |  | |  |  |  |
| T1 | | 53 | | 777777777760771 | | | + | | LAM | 0 | 2 |
|  | | | | | | | - | | NO-LAM | 1 | 0 |
| T1 | | 51 | | 777777777760700 | | | - | | NO-LAM | 1 | 0 |
|  | | | | | | |  | |  |  |  |
| T1 | | 1905 | | 777777777460771 | | | + | | LAM | 0 | 1 |
|  | | | | | | |  | |  |  |  |
| H3 | | 49 | | 777777777720731 | | | - | | NO-LAM | 0 | 1 |
|  | | | | | | |  | |  |  |  |
| H3 | | | 50 | 777777777720771 | | | + | | LAM | 0 | 1 |
|  | | | | | | |  | |  |  |  |
| UNKONWN | | | 1241 | 777777607700771 | | | - | | LAM | 1 | 0 |
|  | | | | | | |  | |  |  |  |
| UNKONWN | | | 2110 | 777777607000771 | | | + | | NO-LAM | 0 | 1 |
|  | | | | | | |  | |  |  |  |
| UNKONWN | | | ORPHAN | 677677606000171 | | | + | | LAM | 2 | 0 |
|  | | | | | | |  | |  |  |  |
| UNKONWN | | | UNKONWN | 777777600000031 | | | + | | LAM | 0 | 1 |
|  | | | | | | |  | |  |  |  |
| UNKONWN | | | UNKONWN | 677777607700371 | | | + | | MIXED | 0 | 1 |
|  | | | | | | |  | |  |  |  |
| UNKONWN | | | UNKONWN | 740001607760771 | | | + | | LAM | 0 | 1 |
|  | | | | | | |  | |  |  |  |
| UNKONWN | | | UNKONWN | 177700607760771 | | | + | | LAM | 0 | 1 |
|  | | | | | | |  | |  |  |  |
| UNKONWN | | | UNKONWN | 777777607740431 | | | + | | NO-LAM | 0 | 1 |
|  | | | | | | |  | |  |  |  |

Legend: + = RD174 pattern; - = WT; +/- = Mixed pattern
